# The effectiveness of Du moxibustion for ankylosing spondylitis: A protocol for systematic review and meta-analysis of randomized clinical trials

**DOI:** 10.1097/MD.0000000000021450

**Published:** 2020-07-31

**Authors:** Shouqiang Huang, Honglian Li, Jun Xiong, Fanghui Hua, Jie Xiang, Yunfeng Jiang

**Affiliations:** a Jiangxi University of Traditional Chinese Medicine; b Haiyang People's Hospital of Shandong Province, Haiyang; c The Affiliated Hospital of Jiangxi University of Traditional Chinese Medicine, Nanchang, P.R. China.

**Keywords:** ankylosing spondylitis, Du moxibustion, protocol, systematic review

## Abstract

**Background:**

Ankylosing spondylitis (AS) is a common progressive autoimmune inflammatory disease. Du moxibustion can effectively treat AS with few adverse reactions. The aim of this protocol is to systematically investigate the effectiveness and safety for management of AS with Du moxibustion.

**Methods:**

Seven relevant databases, namely, PubMed, Cochrane Library, Embase, Chinese Biomedical Literatures Database (CBM), China National Knowledge Infrastructure (CNKI), WangFang Database (WF), Chinese Scientific Journal Database (VIP) will be searched from their inception until May 1st, 2020. All clinical randomized controlled trials containing eligible interventions(s) and outcome(s) will be included, regardless of blinding or publication types. Two reviewers will independently retrieval databases, extract data, and then assess the quality of studies. Data synthesis will be conducted by RevMan 5.3 software. We regard the effective rate, Bath Ankylosing Spondylitis Disease Activity Index (BASDAI), Visual Analogue Scale (VAS) as the primary outcomes, and the secondary outcomes contain C-reactive protein (CRP), erythrocyte sedimentation rate (ESR), finger-to-floor distance (FFD), occiput to wall distance (OWD), and side effects. The result about the curative effect and safety of Du moxibustion for AS will be presented as risk ratio for dichotomous data and mean differences with a 95% confidence interval for continuous data.

**Results:**

The finding will be presented in a journal or related conferences.

**Conclusions:**

This study expects to provide high-quality, evidence-based recommendations on further treatment for clinical guidance.

**PROSPERO registration number:**

CRD42020158727.

## 1 Introduction

Ankylosing spondylitis (AS), a complex autoimmune inflammatory disorder, is characterized by inflammatory back pain and progressive bone fusion of the spine and may involve the arthritis in the hips, shoulders, or peripheral joints.^[1,2]^ In some case, this situation seriously affects the physical function and life quality.

An epidemiological survey has shown that the AS prevalence rates in the United States ranged from 0.2% to 0.55%.^[3]^ In Norway, the average prevalence of AS has been reported to be 1.1% to 1.4%.^[4]^ A Chinese study also described that the average incidence is 0.2% to 0.3% among China.^[5]^ In addition, a study revealed that AS occurs much more in men compared with women, and the prevalence rate of males is 2 to 3 times higher than that of women.^[6–8]^ The concern is that patients with AS always suffer from dysfunction and have a poor quality of life, which bring a heavy burden on patients, families, and society.^[9]^ An economic survey conducted in American has reported that the average direct medical expenses of AS were $2674 in the first year, while the indirect expenses were $4945.^[10]^ Another early report has shown that the yearly mean total costs in Europe as much as Euro 9462 per AS patients.^[11]^ Moreover, AS will increase the risk of malignancy by 14% as well as the prevalence of depression.^[12–14]^

The pathogenesis of AS is quite complex and multifactorial, which has not been fully elucidated so far. In the early years ago, it has been proved that AS is related with the inheritance of HLA allele B27, which might misfold in the ER, causing upregulation of IL-23 in dendritic cells.^[15–17]^ Recently, many studies have highlighted a key role for intestinal dysbiosis in the development of ankylosing spondylitis and suggested that 70% of AS patients accompanied with subclinical intestinal inflammation.^[18]^ Furthermore, another recent study has emphasised the significant of IL-17a/IL-23 cytokine dysregulation in the pathogenesis of AS.^[19]^

Currently, nonsteroidal anti-inflammatory drugs (NSAIDs) are regarded as first-line therapy for the patients with AS according to evidence-based medicine, which works through inhibiting prostaglandin synthase from relieving pain, stiffness and inflammation. Nevertheless, prolonged treatment with these drugs will be associated with side effects, such as adverse cardiovascular and renal effects.^[20]^ Disease-modifying anti-rheumatic drugs (DMARDs) as second-line therapy for AS also play an significant role in increasing the activity index, improving peripheral joint disease, and reducing disease activity.^[21]^ Meanwhile, tumor necrosis factor (TNF) inhibitors can partially block the function of these inflammation pathways, but the expense of these agents may be too expensive so that they are inaccessible for most of AS patients.^[22]^ Therefore, more and more AS patients start to focus on substitution and complementary therapies, such as Chinese herbal medicine, acupuncture, and moxibustion.

Du moxibustion, as a special moxibustion therapy, can effectively improve the symptoms of AS patients, and has been widely used in rheumatic immune system diseases and osteoarthropathy.^[23]^ Du moxibustion refers to the application of moxa, traditional Chinese medicine, or other mediums on the spine of Du vessel, which has the advantages of a wide area of moxibustion, deep penetration of heat, strong temperature, long-lasting effect, and so on.^[23,24]^

The therapy has a 2-way benign regulatory effect, that can effectively regulate the immune imbalance and inflammatory reaction in patients with AS, promote the recovery of joint functional range of motion, and prevent joint stiffness and deformity.^[25–27]^ Several systematic review and meta-analyses published in Chinese have reported that Du moxibustion treatments have curative effect on AS patients.^[28–31]^ At the same time, the use of this alternative therapy can avoid the side effects of conventional oral drugs and reduce the patients’ physical and economic burden.

Although plenty of studies have been performed to examine the effect of Du moxibustion treatments on AS, the evidence supporting its effectiveness is still limited. From the perspective of evidence-based medicine, the participants included in the previous review all were from China, and most of the RCTs lacked rigorous trial design methods and without observation on the long-term effect of treatment results. It also did not provide study protocols ahead of schedule. Thus, we re-evaluate this issue based on the most comprehensive and latest resources of treating AS with Du moxibustion. In this review, we aim to provide reliable evidence for medical practitioners, researchers regarding helping AS patients seek more reasonable treatments.

## 2 Objectives

This study is aimed at summarizing the clinical evidence about the effectiveness and safety of Du moxibustion for AR and provides doctors, patients, policy decision makers with reliable recommendations.

## 3 Methods and analysis

### 3.1 Study registration

The protocol of the present study was recorded in the PROSPERO with the registration number of CRD42020158727, which could obtain from the following URL: http://www.crd.york.ac.uk/PROSPERO/display_record.php?ID=CRD42020158727. We will draft this protocol under the guidelines for systematic review and meta-analysis protocol (PRISMA-P) statement in the Cochrane Hand book,^[32]^ and any changes will be described in our full review.

### 3.2 Inclusion certain for study selection

#### 3.2.1 Types of studies

There is no restriction on blinding or publication type, all randomized clinical trials (RCTs) published in both Chinese and English on Du moxibustion in the treatment of AS can be included.

#### 3.2.2 Types of participants

Participants who meet the diagnostic criteria^[33]^ of ankylosing spondylitis revised by American rheumatology society in 1984 will be included. Secondly, patients in different types of AS can be included, irrespective of sex, race, age marital status, education, economic status.

#### 3.2.3 Types of interventions

The interventions of the treatment group include the single treatment of Du moxibustion or in combination with other conventional therapies (e.g., medication/drugs, Chinese herbs, acupuncture, tuina, and so on). The interventions of the control group comprise of sham moxibustion, placebo, no treatment, and other active therapies, without restrictions of retaining time, course of treatment, and follow-up period. We will select the treatment comparisons as follows:

(1) Du moxibustion contrast with no treatment;
(2) Du moxibustion contrast with placebo or sham moxibustion;
(3) Du moxibustion contrast with other active therapy;
(4) Du moxibustion plus other active therapy contrast with the same active therapy.

#### 3.2.4 Types of outcome measures

##### 3.2.4.1 Primary outcomes

We consider the effective rate, Bath Ankylosing Spondylitis Disease Activity Index (BASDAI), and Visual Analogue Scale (VAS) as the primary outcomes.

##### 3.2.4.2 Secondary outcomes

The secondary outcomes of this review mainly involve the following items:

(1) Physical examination: Finger-to-floor distance (FFD), occiput to wall distance (OWD).
(2) Laboratory examination: C-reactive protein (CRP), erythrocyte sedimentation rate (ESR).
(3) Side effects: The blood and urine routine, liver and kidney function as well as other adverse reactions.

### 3.3 Exclusion certain for study selection

The exclusion certain contains the following items:

(1) Patients with endocrine diseases or other serum negative spondyloarthropathy and rheumatic diseases that may cause osteoporosis.
(2) The literature related to the same study, and duplicated publications.
(3) Unable to get literature for available data or full text through various means.

### 3.4 Search methods identification of studies

Seven relevant databases, namely, PubMed, Cochrane Library, Embase, Chinese Biomedical Literatures Database (CBM), China National Knowledge Infrastructure (CNKI), WangFang Database (WF), Chinese Scientific Journal Database (VIP) will be searched from their inception until May 1, 2020. The search term will be formed with the disease term part (e.g., ankylosing spondylitis OR spondyloarthritis OR spondyloarthropathies OR seronegative spondyloarthropathies), the intervention term part (e.g., Du moxibustion OR long snake moxibustion OR dragon moxibustion), and study type (e.g., randomized controlled trial OR controlled clinical trial OR randomized), and MeSH items and free words will be searched synchronously. The specific search strategy for PubMed is presented in Table 1.

**Table 1:**
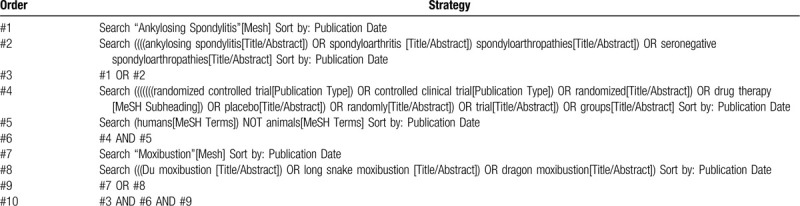
The search strategy for PubMed.

Besides that, to ensure the comprehensiveness of literature retrieval, we will search for eligible ongoing or unpublished trials through the WHO international clinical trials registry platform and the Chinese clinical registry.

### 3.5 Data collection and analysis

#### 3.5.1 Pilot search

In order to reduce the difference in documents selection standards, we will randomly select some documents for a predictive test. By making a discussion about the inconsistencies of our document selection, we will be better able to understand the inclusion and exclusion criteria for each systematic review.

#### 3.5.2 Study selection

**Figure 1:**
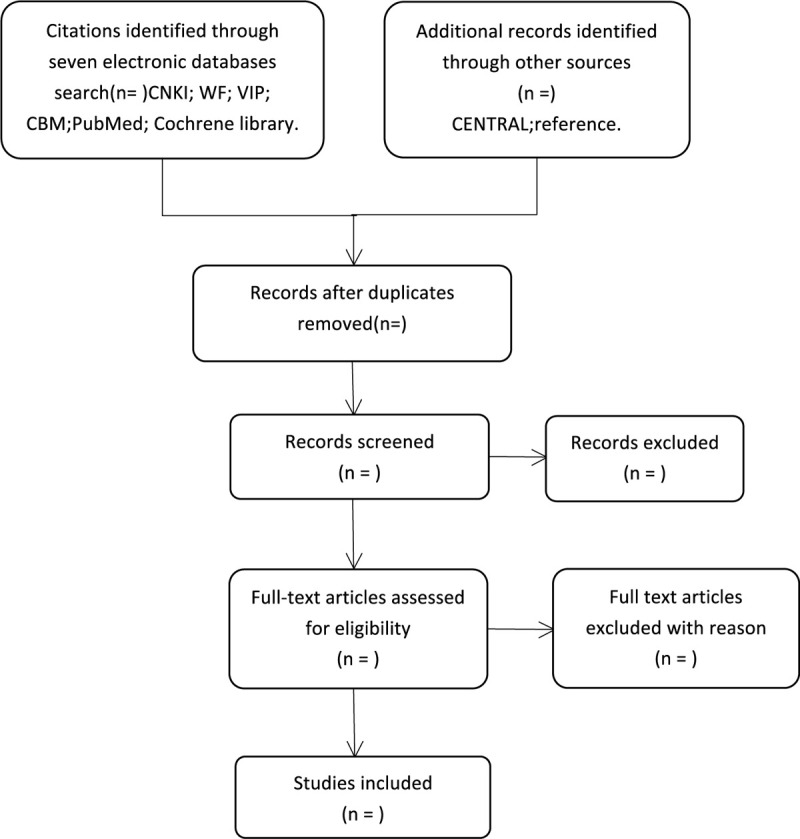
Flowchart of literature selection.

The search results will be imported into NoteExpress 3.2.0 for management. Firstly, the 2 reviewers (HFH and JX) will respectively browse the title and abstract to eliminate some ineligible articles. Then they will read the full text of all potential related research to decide on inclusion. If articles included unclear information or missing data is not enough to determine eligibility, QSH would attempt to contact the original author for details. Under the circumstance of disagreements, discussion or negotiation will be carried out with the third reviewer (FYJ) to reach a consensus. The details of the studies selection will be displayed in a flow chart (Fig. 1).

#### 3.5.3 Data extraction

Two independent reviewers (HFH and JX) will design an extraction form to collect general information, which mainly involves author, publication year, participants, intervention (s), comparison (s), outcome (s), adverse events, as well as some related features in the full text. For documents lacking important information or data, we will contact the first corresponding authors to obtain relevant information. In case of any disagreements, we will try to resolve it through discussion or consensus with the third reviewer (FYJ).

#### 3.5.4 Assessment the risk of bias in studies

The risk of bias tool from Cochrane Reviewer's Handbook 5.0.24^[34]^ will be used for evaluating the methodological quality of the included studies, which will be independently conducted by 2 authors (JX and HFH) from the following 7 aspects: randomly generated sequence number, allocation sequence concealment, blinding of participants and personnel, blinding of outcome assessors, incomplete outcome data, selective outcome reporting and other sources of bias. The trial will be classified into “high risk,” “low risk,” or “unclear risk” for each aspect. The rating results will be cross-checked, and any disagreements will be consulted with the third review author.

#### 3.5.5 Assessment of reporting biases

Two independent review authors will assess the reporting quality to determine whether they meet the criteria specified in the Consolidated Standards of Reporting Trials^[35]^ and Standards for Reporting Interventions in Clinical Trials of Moxibustion checklist.^[36]^ The third review author (FYJ) will be in charge of clarifying issues when disagreements arise.

#### 3.5.6 Measures of curative effect

All statistical analyses will be conducted by Review Manager (RevMan) [Computer program]. (Version 5.3. Copenhagen: The Nordic Cochrane Centre, The Cochrane Collaboration, 2014. Java Vendor: Sun Microsystems Inc). The results of continuous variables will be measured by using weight mean difference (WMD) with its 95% confidence interval (CI), while the results of dichotomous data using risk ratio (RR) and its 95% CI.

#### 3.5.7 Data analysis

The data from parallel-group studies will be collected for analysis. If some studies involve multiple intervention groups, we will combine all related study group and control group into a single group. In the end, every single data for outcome indicators will be gathered for evaluation.

#### 3.5.8 Missing data

For some included studies with vital details of methods and results missing, we will contact the first corresponding author to get specific information by telephone or email. If we can’t obtain that missing information, the analysis will be performed according to the available data. Besides, we will consider the potential impact of missing data for our studies.

#### 3.5.9 Assessment of heterogeneity

Heterogeneity is referred to the difference among studies in the systematic review,^[37]^ which is quantified by the value of *I*^2^. If the *I*^2^ value is <50%, that indicates slight or no statistical heterogeneity in these studies. Once the *I*^2^ value surpasses 50%, it means studies with high heterogeneity, and we will carry out sensitivity analysis or subgroup analysis for finding the possible reasons.

#### 3.5.10 Synthesis of data

The data synthesis will be executed with Review Manager V.5.3 statistical software after unifying the units of each result of different tests. We will select the random-effects model (*I*^2^ ≥ 50%) or fixed-effects model (*I*^2^ < 50%) properly based on the heterogeneity levels. When it came to insufficient RCTs or unidentified significant heterogeneity, we will conduct a narrative, the qualitative summary, or subgroup analysis. At the same time, funnel plots will be employed to assess the publication bias when the included trials >10.

#### 3.5.11 Subgroup analysis

If the necessary data are available, we will carry out subgroup analysis in line with the type of AS, the duration or dosage of Du moxibustion, treatment frequency, and the type of intervention in the control group or the study group.

#### 3.5.12 Sensitivity analysis

If there is significant heterogeneity, a sensitivity analysis will be conducted from the aspects of sample size, the quality of research, methodological elements, and characteristic of research. In the meantime, we will get rid of low-quality or small sample studies to evaluate whether the conclusions are stable. If sensitivity analysis changes the results, we must be more careful in reaching conclusions.

#### 3.5.13 Grading the quality of evidence

The evidence quality from each study will be assessed by 2 independent reviewers using the GRADE instrument.^[38]^ The specific evidence quality will be evaluated according to the 5 aspects (inconsistency, limitations, imprecision, indirectness, and publication bias), it will be rated as 4 levels (high, moderate, low, or very low).^[39]^

## 4 Discussion

Ankylosing spondylitis (AS), a painful and debilitating disease, mainly affects the sacroiliac joints and axial skeleton, causing severe back pain, spinal stiffness. AS is more common in young men with the characteristics of hidden onset, long course of the disease, and high disability rate, which has gradually become a serious public health problem.^[40]^ At present, there is no ideal treatment largely due to the limited knowledge of the pathogenesis. Traditional Chinese Medicine believes that AS belongs to the category of “big hunchback,” and is related to congenital kidney deficiency and acquired pathogenic factors. As a traditional Chinese medicine therapy, Du moxibustion has a unique capability for treating AS. There are an increasing number of clinical trials reports that Du moxibustion is effective in treating ankylosing spondylitis. However, previous studies have some limitations, such as lack of study protocols, without rigorous trial design methods and so on. Hence, we perform this systematic review according to the latest resources in the hope that provide reliable evidence and establish a better approach for treating AS with Du moxibustion.

However, the above protocol still have limitations: we should recognize that our electronic search only involves Chinese and English that may cause Inadequate retrieval; Otherwise, many trials are difficult to implement the blind method in the process of Du moxibustion, which may lead to bias.

## Author contributions

Shouqiang Huang conceived the review protocol and drafted the manuscript. Yunfeng Jiang, Jun Xiong revised the study design. Shouqiang Huang, Honglian Li, Fanghui Hua, Jie Xiang and Yunfeng Jiang participated in the design of the search strategy and data extraction data set. Yunfeng Jiang, Shouqiang Huang and Jun Xiong formed the data synthesis and analysis plan. In practice, Yunfeng Jiang and Shouqiang Huang will monitor each procedure of the review and are responsible for the quality control. All authors have read and approved the publication of the protocol.
